# Genotypic characterization of extended-spectrum beta-lactamase-producing *E. coli* from dogs in northern Germany

**DOI:** 10.1128/spectrum.00087-25

**Published:** 2025-05-22

**Authors:** Marco Werhahn Beining, Sebastian Guenther, Antina Lüebke-Becker, Stefan E. Heiden, Katharina Schaufler, Lothar Kreienbrock, Michael Schwabe

**Affiliations:** 1 Institute of Biometry, Epidemiology and Information Processing, WHO-Collaborating Center for Research and Training in Veterinary Public Health, University of Veterinary Medicine, Hannover, Germany; 2 Pharmaceutical Biology, Institute of Pharmacy, University of Greifswald 26552, Greifswald, Germany; 3 Center for Infection Medicine, Institute of Microbiology and Epizootics, Freie Universität Berlin 9166, Berlin, Germany; 4 Epidemiology and Ecology of Antimicrobial Resistance, Helmholtz Institute for One Health, Helmholtz Center for Infection Research HZI, Greifswald, Germany; 5 University Medicine Greifswald 60634, Greifswald, Germany; University of Brescia, Brescia, Italy

**Keywords:** antibiotic resistance, ESBL* Escherichia coli*, beta-lactamase, drug resistance

## Abstract

Antimicrobial resistance (AMR) is a growing concern in veterinary and public health, with extended-spectrum beta-lactamase (ESBL)-producing *Escherichia coli* playing a significant role. This study examined 1,000 healthy and sick dogs from a veterinary clinic in northern Germany and identified 85 ESBL-producing *E. coli*. Whole-genome sequencing of these isolates revealed seven phylogroups. A (38.8%) and B1 (32.9%) were the most common. Multilocus sequence typing identified 42 sequence types (STs), with the globally occurring lineages ST744 and ST10 being predominant. Single nucleotide polymorphism analysis showed the clonal circulation of ST744 among dogs in shared environments, such as households or breeders, whereas ST10 isolates displayed greater genetic diversity. ST131, a pathogenic international high-risk clonal lineage often associated with humans, was assigned to one isolate. Virulence-associated genes (VAGs) were abundant across the isolates, with siderophore systems, biofilm formation, and adherence traits being prominent. All isolates carried enterobactin genes with additional siderophore systems, such as yersiniabactin and aerobactin, present in 36 isolates. The highest number of VAGs (25) was observed in isolates belonging to the pathogenic lineages ST648 and ST405. Sixty-nine percent of the isolates were multidrug-resistant, carrying resistance genes for three or more antibiotic classes, with beta-lactam, aminoglycoside, and tetracycline resistance being the most frequent. This study highlights globally occurring *E. coli* lineages in companion animals and the role of close contact environments in their dissemination. Although dog-to-human transmission was not investigated in this study, these findings support the need for a One Health approach to address AMR, emphasizing the interconnected health of humans, animals, and the environment.

**IMPORTANCE:**

This study demonstrated the presence of globally significant *Escherichia coli* lineages in dogs and highlighted the impact of close-contact environments, such as households and breeders, on their spread. Many of the isolates exhibited genetic multidrug resistance and virulence features, posing challenges for effective treatment and control. These findings emphasize the interconnected nature of human, animal, and environmental health, underlining the need for a One Health approach to address antimicrobial resistance.

## INTRODUCTION

*Escherichia coli* is a highly diverse bacterial species comprising various pathotypes, serotypes, and phylogenetic types. It is a common and ubiquitous component of the intestinal microbiome, typically colonizing mammals and birds as commensal organisms (1). However, *E. coli* can also cause severe diseases, including diarrhea, sepsis, urogenital infections, and wound infections (2).

Within pathogenic *E. coli*, two types can be distinguished. While intra-intestinal pathogenic *E. coli* (InPEC) causes various enteric infections, extra-intestinal pathogenic *E. coli* (ExPEC) isolates are responsible for diseases outside the intestinal tract such as urinary tract infections and meningitis (3). ExPEC strains, in particular, are frequently linked to antibiotic resistance genes, including extended-spectrum beta-lactamases (ESBLs), which can degrade third-generation cephalosporins. These resistance genes are commonly carried on plasmids (4). In addition, these pathogens are often resistant to multiple classes of antimicrobials and are therefore considered multidrug resistant (MDR).

MDR *E. coli* isolates occur not only in human and veterinary clinical settings but also in the community, pets, and the environment (5, 6). In addition, so-called international high-risk clonal lineages, such as sequence types (STs) 131, 648, and 410, occur frequently in different ecologies and hosts. They are characterized by a combination of MDR, with high fitness and virulence properties (7). The One Health framework acknowledges the interconnectedness between human, animal, and environmental health. This emphasizes the need for a unified approach to prevent the emergence and transmission of antibiotic resistance. Key aspects of this strategy include resistance transmission pathways, shared environments, antimicrobial usage, foodborne transmission, and environmental contamination.

Several studies have highlighted that dogs can act as reservoirs for ESBL-producing *E. coli*, emphasizing their potential role in the persistence and spread of antimicrobial resistance (AMR) (8, 9). This is of particular concern because of the close contact between dogs and humans, which creates opportunities for the bidirectional transmission of resistant bacteria. Published studies already provided evidence that ESBL-producing *E. coli* strains isolated from dogs are often genetically similar to those found in human owners, suggesting a shared microbiological environment. For example, strains that harbor resistance determinants, such as *bla*_CTX-M_ genes, have been identified in both dogs and their owners, pointing to direct or indirect pathways of transmission, and transmission between owners and dogs is possible (10, 11)

This study involved a comprehensive investigation of a large cohort of dogs within a specific timeframe and geographic area accompanied by detailed contextual information. The primary aim was to analyze resistance distributions and establish a robust database for individual clonal lineages. These findings not only serve as a resource for future comparative studies but also provide critical insights for developing targeted strategies to combat MDR pathogens.

## RESULTS AND DISCUSSION

### The presence of ESBL-producing *E. coli* in dogs

This study examined 1,000 dogs, comprising patients with and without clinical symptoms, from a veterinary clinic in Germany. Rectal swabs were collected in 2017 to screen for ESBL-producing *E. coli*, resulting in 85 positive isolates. Out of the 85 isolates, 16 were sampled from sick dogs. An earlier epidemiological analysis focusing on various factors associated with ESBL positivity (e.g., feeding habits and lifestyle) was published (12).

**Fig 1:**
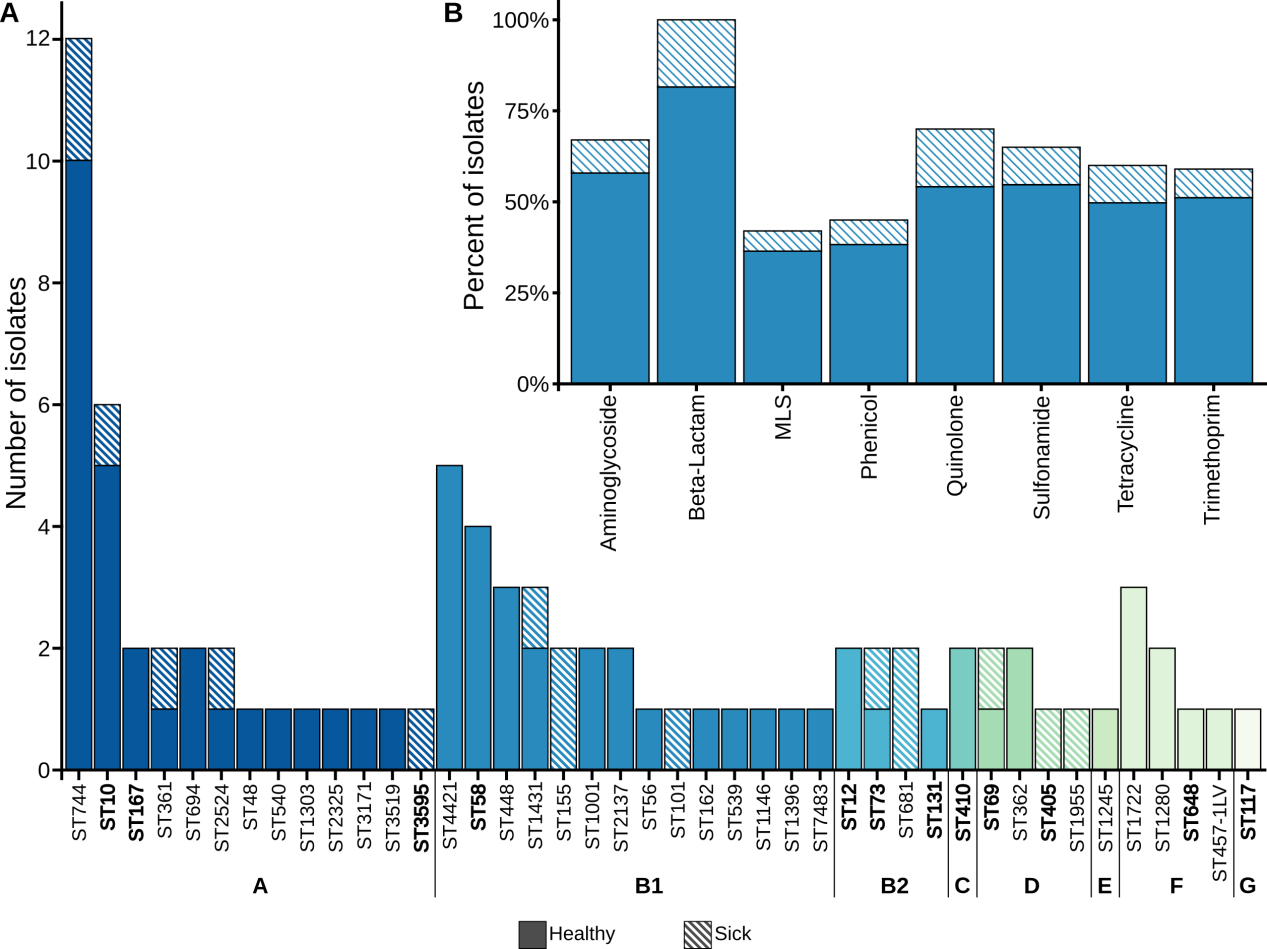
(A) Distribution of STs and phylogroups among ESBL-producing *E. coli*. Forty-two different STs were identified. Pandemic sequence types of *E. coli* are highlighted in bold. (**B**) Percent of isolates showing genotypic resistances to selected antibiotic classes, based on AMRFinderPlus results (MLS: macrolides, lincosamides, and streptogramines).

Bioinformatics analysis revealed that the 85 *E. coli* isolates were assigned to seven phylogroups: A, B1, B2, C, D, F, and G. Phylogroups A (38.8%) and B1 (32.9%) were the most prevalent, whereas phylogroup C was the least common (two isolates; Fig. 1A). Notably, only seven isolates belonged to phylogroup D, which was associated with high virulence and severe infections. Of these seven isolates, only three dogs from which they were derived exhibited acute diseases. Additionally, one isolate belonged to the recently described phylogroup G. It is worth noting that phylogroups A and B1 are generally linked to commensal strains (13) and InPEC infections. Furthermore, phylogroup A isolates are frequently found in human stool samples (14).

Multilocus sequence typing identified 41 distinct STs among the isolates, and one isolate was assigned as an allelic variant of ST457 (ST457-1LV). Several globally recognized high-risk clonal lineages, including ST131 (*n* = 1), ST410 (*n* = 2), and ST648 (*n* = 1), have been identified (15–18). The abundance of high-risk clonal lineages in sick dogs is not significant compared to healthy dogs. The most common sequence types were ST744 (*n* = 12) and ST10 (*n* = 6; Fig. 1A). ST744, which belongs to phylogroup A, has been widely reported in humans, wildlife, dogs, and environmental sources (19–24). This clone has been found in thermotolerant *E. coli* strains isolated from ready-to-eat street food (25). The second most common type, ST10, is frequently associated with commensal intestinal *E. coli* colonization (26).

The distribution of ST744 and ST10 in this study aligns with the findings of Boehmer et al. (20), who reported a high prevalence of ST744 followed by ST10 in fecal samples from military dogs collected over a 1-year period. This is not entirely surprising, given that rectal swabs directly target the intestinal microbial community, especially as ST10 is frequently associated with commensal strains of *E. coli*. However, the notable abundance of ST744, which has also been linked to its global occurrence and diverse ecological niches, raises questions about its persistence and potential for transmission within and beyond canine populations. This also highlights the importance of monitoring the dynamics of specific STs in various host environments and underscores the need for comparative studies to explore the factors contributing to the success of these clones in intestinal and extraintestinal contexts.

The ST410 and ST648 sequence types, only found in healthy dogs in this study, have been previously identified in humans, domestic animals, and environmental samples (27). Schaufler et al. (2016b) reported an ESBL-producing *E. coli* clone of ST410 in environmental dog feces as well as in a clinical case involving a dog, whereas Timofte et al. (2016) detected *E. coli* ST410 in a dog with septic peritonitis.

Although ST648 was identified only once in our study, it has been recognized as an intercontinental high-risk clonal lineage that is strongly linked to human infectious diseases (17, 28, 29). Given its global distribution and significance as a major international high-risk clone (30), the detection of only one ST131 isolate (healthy dog) in this study was unexpected. Interestingly, Boehmer et al. (20) did not report ST131 in their analysis (20). The low prevalence of ST131 in our findings could potentially be explained by its strong association with humans, as this clone is primarily recognized as a major cause of human infections. Furthermore, >50% of the pets in this study were presented for prophylactic reasons while carrying ESBL-producing *E. coli*, which may have contributed to the low detection rate. Additionally, the limited adaptation of ST131 to canine hosts (31) may explain its rarity in this cohort. First identified in 2008, ST131 has become the dominant ExPEC sequence type globally and is frequently associated with the CTX-M-15 variant of ESBL enzymes (26, 30). These enzymes have been recognized for their potential to induce therapeutic limitations, as reported as early as 2010 (32). ESBL-producing *E. coli* is often detected in contaminated environmental sources, posing a transmission risk to humans either through direct contact or human-to-animal transmission routes (33).

### Phylogenetic relationships

**Fig 2:**
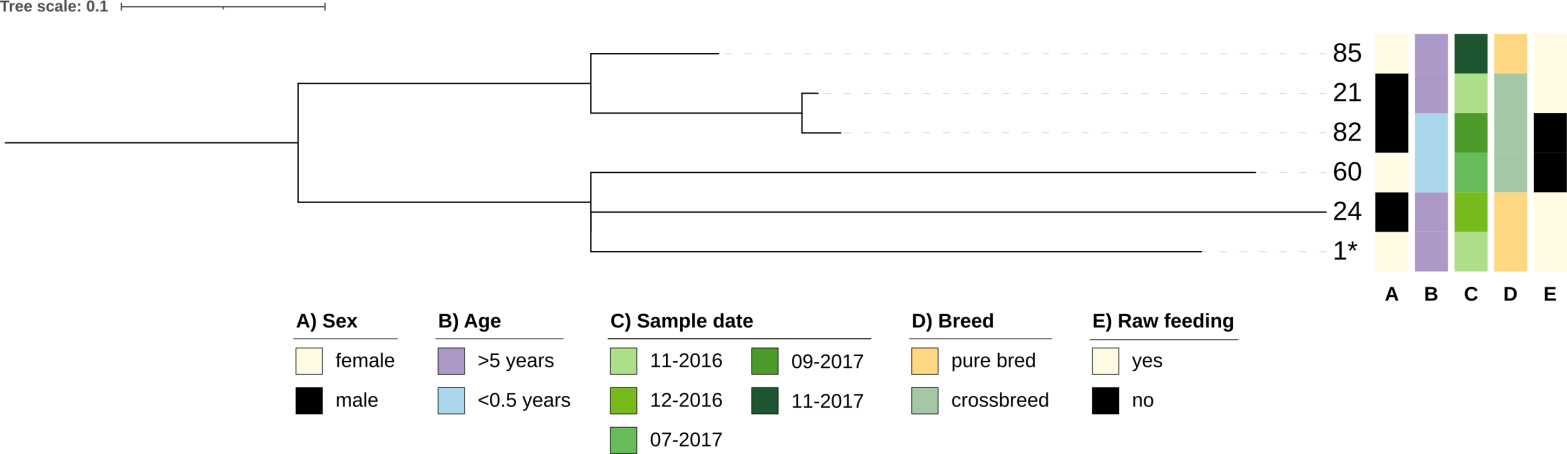
Core SNP phylogeny of ST744. The tree was inferred with an ML-based approach and is based on a core SNP alignment created with snippy (core SNP sites: 195; *: isolate 7 used as reference) and visualized with iTOL. Clusters were manually assigned.

**Fig 3:**
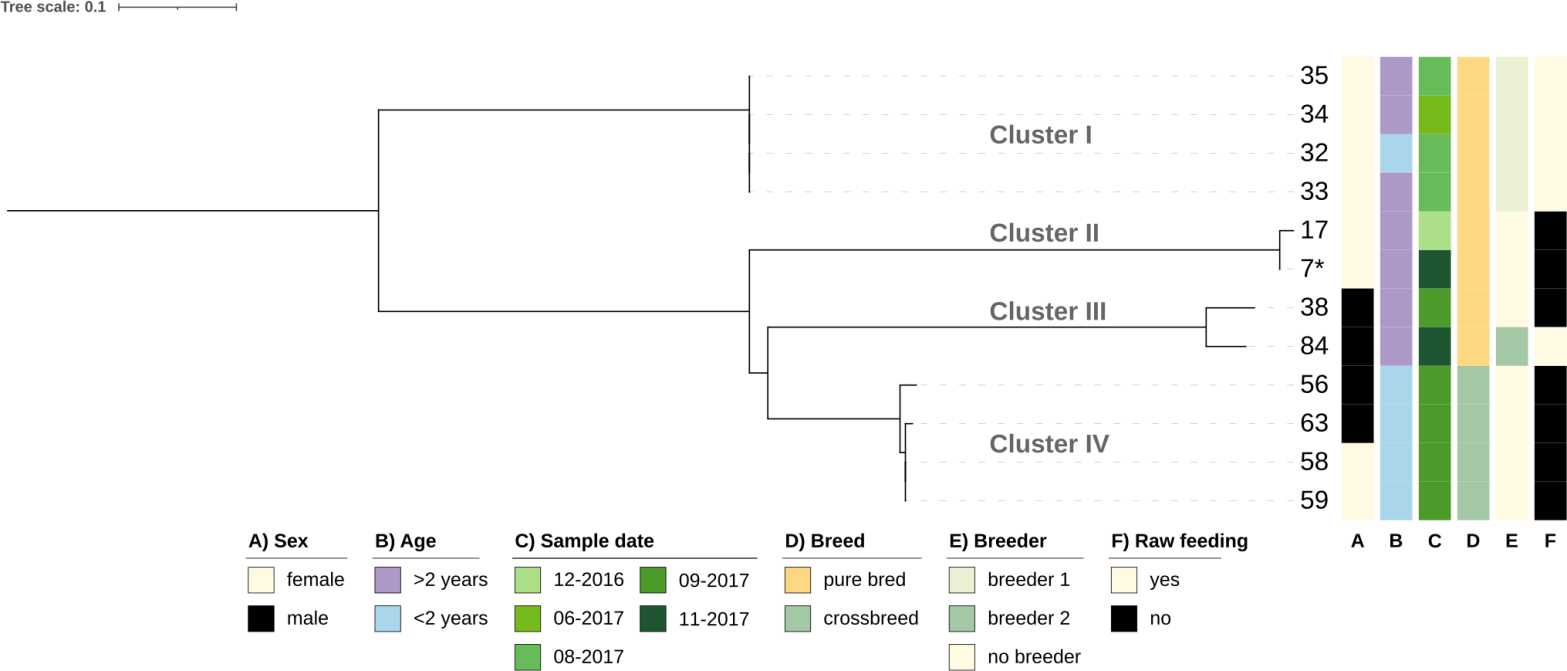
Core SNP phylogeny of ST10. The tree was inferred with an ML-based approach and is based on a core SNP alignment created with snippy (core SNP sites: 2,383; *: isolate 1 was used as reference) and visualized with iTOL. Clusters were manually assigned.

Given the high prevalence of ST744 and ST10 in our sample set, we conducted an in-depth core-single nucleotide polymorphism (core-SNP) phylogenetic analysis. This approach was employed to evaluate potential clonal relationships and investigate the transmission dynamics of these isolates, providing insights into their evolutionary connections and possible routes of dissemination (Fig. 2 and 3).

Four distinct clusters were identified in the phylogenetic tree of ST744. Two of these clusters, clusters I and IV, each contained four samples, while clusters II and III contained two samples each. The core SNP differences ranged from a maximum of 114 to a minimum of 0 (isolates within cluster I). Notably, cluster I of ST744 included isolates from dogs originating from the same breeder (Fig. 2), indicating the circulation of this clonal lineage among animals living in the same household (34, 35) and may be influenced by shared litter boxes and caregivers (7, 36, 37). Multi-dog households and contact with dogs with puppies were also statistically significant risk factors for ESBL-producing *E. coli* carriage (12). We previously described an association between feeding (raw meat and commercial food) and carriage of ESBL-producing *E. coli*, and Leonard et al. (38) reported that raw diet and feeding raw products are other risk factors for colonization with ESBL-producing *E. coli* (38). Interestingly, only five of the 12 dogs carrying ESBL-positive *E. coli* ST744 were fed raw meat. Based on the same data set, we identified feeding raw meat as one of seven factors associated with the occurrence of ESBL-producing *E. coli* (12). This does not appear to apply to the spread of ST744.

ST10 is a globally distributed lineage frequently reported in studies worldwide—particularly in colonization isolates—and is generally classified as a commensal lineage (26), but it is also known to carry resistance genes. While these genes may be of clinical importance, this lineage often lacks the virulence-associated factors necessary for causing severe diseases (39). In this study, ST10 was the second most abundant ST; however, its presence within the sampled dogs did not suggest clonal circulation, as the dogs were owned by different individuals, had no shared origin or history, and were kept under unrelated conditions. This hypothesis was supported by the results of the SNP analysis. The maximum number of core SNPs observed (1,177 between isolates 60 and 82) was approximately 10 times greater than that observed for ST744, indicating significant genetic diversity. In addition, no distinct clusters were identified in the phylogenetic analysis. The small number of core SNPs (75 between isolates 21 and 82) further highlights the absence of close genetic relationships, confirming that the ST10 isolates in this study likely represent unrelated strains rather than the transmission of a single clonal lineage.

### Resistance profiles of the isolated strains

Overall, all 85 isolates tested positive for β-lactamase-encoding resistance genes. Interestingly, no extended-spectrum beta-lactam genes were predicted for eight isolates. Based on phenotypic screening, we would expect every strain to carry one or more ESBL genes. Among the ESBL-positive isolates, the most common ESBL gene was *bla*_CTX-M-15_ (38/85), followed by *bla*_CTX-M-1_ (19/85) and *bla*_CTX-M-14_ (13/85). One of the isolates (isolate 28) carried two CTX-M family genes simultaneously (*bla*_CTX-M-15_ and *bla*_CTX-M-8_). This is in line with previous findings that *bla*_CTX-M-15_ is the most abundant ESBL gene, followed by *bla*_CTX-M-14_, in *Enterobacterales* worldwide (40). Considering the canine population, both *bla*_CTX-M-15_ and *bla*_CTX-M-11_ were the most commonly detected genes in Germany (20) and other countries (41, 42). In addition, *bla*_CTX-M-1_ is not uncommon and has been frequently reported previously (43, 44). Only one of the isolates carried an ESBL gene outside the CTX-M family, harboring the *bla*_CMY-2_ gene. Among the 85 isolates, resistance genes associated with aminoglycosides were identified in 57 isolates, and tetracycline resistance genes were found in 51 isolates (Fig. 1B). The rarest identified resistance genes were those linked to resistance against macrolides and lincosamides (29 out of 85 isolates).

Of the analyzed isolates, 69% (59 of 85) carried antibiotic resistance genes against three or more antibiotic classes listed by Magiorakos et al. (45) for *Enterobacteriaceae* and were therefore classified as MDR (45). The comparison of resistance genes between isolates from sick and healthy animals did not reveal any statistically significant differences (*P* = 0.14). Interestingly, when comparing isolates grouped into commonly pathogenic phylogroups (B2, D, and F) with isolates from commonly apathogenic phylogroups (A, B1, C, and E) (46), a significantly higher number of resistance genes was found in isolates from the commensal phylogroups (*P* = 0.02, CI: 0.36–6.34; Fig. S1A and B). However, it is important to note that the presence of resistance genes does not necessarily equate to their phenotypic expression or penetrance, which depends on factors such as gene regulation, expression levels, and environmental conditions. This highlights the importance of complementing genomic data with phenotypic resistance testing for a more comprehensive understanding of AMR.

Owing to its broad spectrum and relatively low cost, tetracycline is widely used in the prophylaxis and therapy of human and animal infections, which may lead to a higher incidence of resistant bacteria (47). Tetracycline resistance genes have been frequently described in *E. coli* originating from cattle and pigs (48, 49) as well as in meat originating from pork, beef, chicken, and turkey (50).

Aminoglycoside and sulfonamide resistance genes have previously been detected in isolates from dogs with severe *E. coli* infections, resulting in hemorrhagic enteritis associated with renal involvement and cerebral vasoconstriction (51).

None of the isolates carried genes associated with resistance to colistin, carbapenems, fosfomycin, fusidic acid, nitroimidazole, oxazolidinone, or rifampicin.

### Virulence profiles of the isolated strains

Several studies have reported that the combination of MDR and high virulence is an important feature of high-risk clonal lineages. Therefore, we also analyzed the presence of virulence-associated genes (VAGs) in each isolate (16, 52, 53), regardless of health status (healthy: 69 and sick: 16). A recent study by Falodun et al. on virulent *E. coli* from feces of healthy pet dogs identified numerous virulence-associated genes, including those related to bacterial iron metabolism, iron acquisition, and biosynthesis of siderophores such as yersiniabactin and aerobactin (54). In our study, all isolates were positive for the enterobactin operon (*entABCDEFS* and *fepABSDG+fes*), whereas 36 isolates carried at least one additional siderophore system. Yersiniabactin (*ybtPQ*) was the most prevalent (*n* = 25), followed by aerobactin (*iutA* and *iucABCD*) in 24 isolates and salmochelin (*iroBCDEN*) in 18 isolates. Interestingly, 26 of these 36 isolates carried two additional siderophore systems, with yersiniabactin and aerobactin being the most common combinations (9/36). Five isolates carried all three additional systems. Notably, all ST10 isolates were negative for additional siderophore systems, except isolate 82, which harbored aerobactin and salmochelin.

Other VAGs associated with key virulence traits were widely distributed among isolates. Genes related to phagocytosis (*ugd*, detected in 84/85 isolates), biofilm formation (*csgABCDEFG*, present in 85/85 isolates), motility (*flgBCDFGHI* and *che*, in 85/85 isolates), invasion (*ompA*, in 85/85 isolates), and toxin secretion (*hlyA*, in 85/85 isolates). In terms of adherence-related genes that are crucial for colonization and invasion, *ecp* (*n* = 82), *fdeC* (*n* = 79), *htpB* (*n* = 85), and *ilpA* (*n* = 85) were the most abundant. However, P fimbriae genes (*papG* to *papCD*) were found in fewer isolates, ranging from 1 to 20 strains. The most abundant VAGs in this study were adherence (e.g., common pilus, FdeC adhesins, Hsp60 (*htpB*), and intim-like protein [*SinH*]) and invasion (e.g., outer membrane protein A), which can enhance the potential for direct host infection. The highest number of VAGs (26) was detected in isolates belonging to the high-risk clonal lineages ST648 and ST405, both of which were positive for *bla*_CTX-M-15_. No significant difference was observed in the number of VAGs between isolates from healthy and diseased animals. However, isolates grouped into commonly pathogenic phylogroups exhibited a significantly higher number of VAGs compared to those from apathogenic phylogroups (*P* = 1.66 × 10^−16^, CI: 4.31–6.14; Fig. S1C and D).

The ST744 isolates carried 16–18 VAGs, which is about the average number of VAG per clone in our study (18.6 VAG/clone). Within ST744, 12 isolates shared 14 core VAGs, but only one isolate carried P fimbriae (*fim*), and seven isolates carried the capsule synthesis regulatory gene (*rcsAB*).

A recent study from Tunisia (55) found three times more ESBL-producing *E. coli* (26.6%) compared to our study, but this could be due to the fact that they examined diarrheic dogs, whereas we included dogs regardless of their health status. Another Tunisian study detected numerous VAGs related to adhesins, toxins, and invasion factors commonly associated with ExPEC, such as *fim* (type I fimbriae), *pap* (P fimbriae), and *traT* (plasmid transfer protein), which are implicated in urinary tract infections, sepsis, and neonatal meningitis. Similarly, studies from the USA on cefotaxime-resistant *E. coli* isolates from dogs identified *fimH* and *uidA* as ubiquitous VAGs, along with other ExPEC-associated genes, such as *traT* (50%), *fyuA* (39%), *ompT* (37%), *iroN* (30%), and *maIX* (26%) (56).

Overall, our genomic analysis revealed that ESBL-producing isolates carry a range of virulence-associated genes in addition to antimicrobial resistance (with no correlation between the number of ARGs and VAGs), thereby likely enhancing their ability to cause infections and survive in various environments. The ability to form biofilms, for example, may confer a protective advantage under resource-limited or hostile conditions, such as exposure to antibiotics, UV radiation, or low temperatures (57) but can also contribute to the pathogenicity (58).

The importance of metal uptake, particularly iron acquisition, in bacterial survival is also evident because these systems are critical in metal-limited environments. The presence of virulence factors commonly associated with UPEC strains, which are often located on mobile genetic elements, further underscores the potential for horizontal gene transfer and enhanced pathogenicity.

These findings are consistent with the results of a large meta-analysis examining ESBL-producing *E. coli* in dogs across five continents, which reported a global prevalence of 6.87% and a high diversity of virulence and resistance genes. A meta-analysis also highlighted Europe as a region with particularly high genetic diversity among ESBL genes and *E. coli* clonal lineages (59).

### Conclusions

Our findings highlight the role of close-contact environments, such as households and breeders, in facilitating the spread of globally relevant *E. coli* lineages among dogs. The identification of virulence-associated genes alongside AMR determinants suggests that these bacteria possess traits that enhance their persistence and pathogenic potential. While direct transmission between dogs and humans was not investigated, the presence of similar lineages across species reinforces the need for continuous surveillance at the animal-human interface. These results contribute to the growing recognition of the One Health perspective as a crucial framework for understanding and addressing antimicrobial resistance in a holistic manner.

## MATERIALS AND METHODS

In this study, we examined 1,000 rectal swabs obtained from healthy and diseased dogs from a veterinary clinic in northern Germany in 2017. All dogs were examined for health status, and a questionnaire was completed by the owner.

Rectal swabs (Amies medium with charcoal; Mastaswab, Mast Diagnostica Reinfeld, Germany) were used for sample collection. All swabs were stored at 8°C until further use. Swabs were sent to the Institute of Microbiology and Epizootics, Center for Infection Medicine, Free University Berlin, Germany, for bacterial isolation and identification.

After streaking on CHROMagar plates containing cefotaxime (2 µg/mL; Mast Diagnostica, Reinfeld, Germany), samples were incubated for 24 h, and putative *E. coli* colonies were subcultured until pure cultures were obtained. Subsequent species identification with pure colonies and determination of phenotypic resistance were performed using MALDI-TOF MS (Bruker). In accordance with the CLSI guidelines, confirmatory tests were performed to differentiate ESBL from AmpC (ampicillinase) producers (60).

ESBL-producing isolates with completed questionnaires (89 in total) were subjected to whole-genome sequencing on an Illumina MiSeq/NovaSeq 6000 (GATCEurofins-9).

Raw reads were quality-trimmed, adapter-trimmed, and contaminant-filtered using BBDuk from BBTools (v. 38.90; https://sourceforge.net/projects/bbmap/). After *de novo* assembly using shovill/SPAdes (v. 1.1.0; https://github.com/tseemann/shovill/), draft genomes were polished by mapping all trimmed reads back to the contigs using bwa (v. 0.7.17-r1188, https://github.com/lh3/bwa) (61) and calling variants using Pilon (v. 1.23; https://github.com/broadinstitute/pilon) (62). ST and antibiotic resistance/virulence gene detection were carried out using mlst (v. 2.19.0; https://github.com/tseemann/mlst/), abricate (v. 1.0.0; https://github.com/tseemann/abricate/), and AMRFinderPlus (v. 3.12.8) (63). All isolates were analyzed using CheckM (v. 1.1.3) (64). We inferred a core SNP phylogeny for isolates of sequence types 744 and 10 using snippy (v. 4.6.0; https://github.com/tseemann/snippy). Alignments were filtered for recombination using Gubbins (v. 3.2.1; https://github.com/sanger-pathogens/gubbins/), and core SNPs were extracted using snp-sites (v. 2.5.1; ST744 core SNP sites: 195; ST10 core SNP sites: 2,383) (65). A maximum likelihood tree was constructed with RAxML-NG (v. 1.1.0) using GTR + G. The best-scoring maximum likelihood tree was midpoint-rooted and visualized using iTOL (https://itol.embl.de/) with metadata. Welsh two-sample *t*-test analysis for statistical significance was performed in R.

## Data Availability

The data for this study have been deposited in the European Nucleotide Archive (ENA) at EMBL-EBI under accession number PRJEB83102 (https://www.ebi.ac.uk/ena/browser/view/PRJEB83102).

## SUPPLEMENTAL MATERIAL

Supplemental material spectrum.00087-25-s0001.docx Table S1; Fig. S1.

ASM does not own the copyrights to Supplemental Material that may be linked to, or accessed through, an article. The authors have granted ASM a non-exclusive, world-wide license to publish the Supplemental Material files. Please contact the corresponding author directly for reuse.
