# Supplementary material for: Genotypic characterization of extended-spectrum beta-lactamase-producing E. coli from dogs in northern Germany
Source: Microbiol Spectr. 2025 May 22;13(7):e00087-25. doi: 10.1128/spectrum.00087-25 (PMC12211048; doi:10.1128/spectrum.00087-25)
Supplement: Supplemental material — Table S1; Fig. S1. [file spectrum.00087-25-s0001.docx]

Table S1: Metadata for ESBL positive isolates. Phylogroup category was assigned based on Bok, E. *et al.* K. Extended Phylogeny and Extraintestinal Virulence Potential of Commensal *Escherichia coli* from Piglets and Sows. Int. J. Environ. Res. Public Health **2020**, 17, 366. <https://doi.org/10.3390/ijerph17010366>“ and “Tenaillon, O. *et al.* The population genetics of commensal *Escherichia coli*. *Nat Rev Microbiol* **8**, 207–217 (2010). https://doi.org/10.1038/nrmicro2298”.

| **Isolate name** | **Sampling data** | **Sampling location** | **Health status** | **Feeding raw meat** | **Sequence type** | **Phylogroup** | **Phylogroup category** |
| --- | --- | --- | --- | --- | --- | --- | --- |
| **1** | 11-Nov-16 | Sottrum; Germany | Healthy | Yes | 10 | A | commensal_PG |
| **2** | 14-Nov-16 | Sottrum; Germany | Sick | Yes | 3595 | A | commensal_PG |
| **3** | 29-Nov-16 | Sottrum; Germany | Sick | Yes | 1955 | D | pathogenic_PG |
| **4** | 22-Nov-16 | Sottrum; Germany | Sick | No | 1431 | B1 | commensal_PG |
| **5** | 16-Nov-17 | Sottrum; Germany | Healthy | No | 3171 | A | commensal_PG |
| **6** | 03-Dec-16 | Sottrum; Germany | Healthy | Yes | 448 | B1 | commensal_PG |
| **7** | 03-Dec-16 | Sottrum; Germany | Healthy | No | 744 | A | commensal_PG |
| **8** | 03-Dec-16 | Sottrum; Germany | Healthy | No | 448 | B1 | commensal_PG |
| **9** | 03-Dec-16 | Sottrum; Germany | Healthy | No | 448 | B1 | commensal_PG |
| **10** | 21-Nov-16 | Sottrum; Germany | Healthy | Yes | 48 | A | commensal_PG |
| **11** | 12-Dec-16 | Sottrum; Germany | Healthy | No | 131 | B2 | pathogenic_PG |
| **12** | 21-Nov-16 | Sottrum; Germany | Sick | Yes | 69 | D | pathogenic_PG |
| **13** | 18-Nov-16 | Sottrum; Germany | Healthy | Yes | 56 | B1 | commensal_PG |
| **14** | 16-Nov-16 | Sottrum; Germany | Healthy | No | 1431 | B1 | commensal_PG |
| **15** | 28-Apr-17 | Sottrum; Germany | Healthy | Yes | 410 | C | commensal_PG |
| **16** | 07-Nov-16 | Sottrum; Germany | Sick | No | 681 | B2 | pathogenic_PG |
| **17** | 06-Dec-16 | Sottrum; Germany | Sick | No | 744 | A | commensal_PG |
| **18** | 03-Nov-16 | Sottrum; Germany | Sick | Yes | 681 | B2 | pathogenic_PG |
| **19** | 25-Oct-16 | Sottrum; Germany | Healthy | Yes | 410 | C | commensal_PG |
| **20** | 21-Oct-16 | Sottrum; Germany | Healthy | No | 539 | B1 | commensal_PG |
| **21** | 26-Nov-16 | Sottrum; Germany | Sick | Yes | 10 | A | commensal_PG |
| **22** | 26-Nov-16 | Sottrum; Germany | Healthy | No | 2524 | A | commensal_PG |
| **23** | 12-Jul-17 | Sottrum; Germany | Healthy | No | 694 | A | commensal_PG |
| **24** | 13-Dec-16 | Sottrum; Germany | Healthy | Yes | 10 | A | commensal_PG |
| **25** | 15-Dec-16 | Sottrum; Germany | Sick | Yes | 361 | A | commensal_PG |
| **26** | 02-Dec-16 | Sottrum; Germany | Sick | No | 2524 | A | commensal_PG |
| **27** | 01-Dec-16 | Sottrum; Germany | Healthy | Yes | 1431 | B1 | commensal_PG |
| **28** | 05-Dec-16 | Sottrum; Germany | Sick | No | 101 | B1 | commensal_PG |
| **29** | 09-Aug-17 | Sottrum; Germany | Healthy | Yes | 694 | A | commensal_PG |
| **30** | 08-Dec-16 | Sottrum; Germany | Healthy | No | - | F | pathogenic_PG |
| **31** | 23-Nov-16 | Sottrum; Germany | Healthy | No | 69 | D | pathogenic_PG |
| **32** | 20-Aug-17 | Sottrum; Germany | Healthy | Yes | 744 | A | commensal_PG |
| **33** | 03-Aug-17 | Sottrum; Germany | Healthy | Yes | 744 | A | commensal_PG |
| **34** | 23-Jun-17 | Sottrum; Germany | Healthy | Yes | 744 | A | commensal_PG |
| **35** | 19-Aug-17 | Sottrum; Germany | Healthy | Yes | 744 | A | commensal_PG |
| **36** | 11-Aug-17 | Sottrum; Germany | Healthy | No | 117 | G | pathogenic_PG |
| **37** | 30-Nov-17 | Sottrum; Germany | Healthy | No | 12 | B2 | pathogenic_PG |
| **38** | 12-Sep-17 | Sottrum; Germany | Sick | No | 744 | A | commensal_PG |
| **39** | 05-Mar-17 | Sottrum; Germany | Healthy | No | 73 | B2 | pathogenic_PG |
| **40** | 11-Jul-17 | Sottrum; Germany | Healthy | Yes | 1303 | A | commensal_PG |
| **41** | 31-Mar-17 | Sottrum; Germany | Healthy | Yes | 1280 | F | pathogenic_PG |
| **42** | 09-Nov-17 | Sottrum; Germany | Healthy | No | 167 | A | commensal_PG |
| **43** | 24-Jan-17 | Sottrum; Germany | Healthy | Yes | 12 | B2 | pathogenic_PG |
| **44** | 12-Jan-17 | Sottrum; Germany | Healthy | Yes | 58 | B1 | commensal_PG |
| **45** | 03-Aug-17 | Sottrum; Germany | Healthy | Yes | 3519 | A | commensal_PG |
| **46** | 21-Nov-16 | Sottrum; Germany | Healthy | Yes | 361 | A | commensal_PG |
| **47** | 19-Feb-17 | Sottrum; Germany | Healthy | Yes | 167 | A | commensal_PG |
| **48** | 21-Dec-16 | Sottrum; Germany | Healthy | Yes | 540 | A | commensal_PG |
| **49** | 23-Apr-17 | Sottrum; Germany | Healthy | Yes | 58 | B1 | commensal_PG |
| **50** | 22-Jun-17 | Sottrum; Germany | Healthy | Yes | 648 | F | pathogenic_PG |
| **51** | 20-Jan-17 | Sottrum; Germany | Sick | Yes | 155 | B1 | commensal_PG |
| **52** | 31-Mar-17 | Sottrum; Germany | Healthy | No | 162 | B1 | commensal_PG |
| **53** | 24-Jan-17 | Sottrum; Germany | Healthy | No | 58 | B1 | commensal_PG |
| **54** | 06-Feb-17 | Sottrum; Germany | Healthy | Yes | 7483 | B1 | commensal_PG |
| **55** | 05-Sep-17 | Sottrum; Germany | Healthy | No | 2137 | B1 | commensal_PG |
| **56** | 05-Sep-17 | Sottrum; Germany | Healthy | No | 744 | A | commensal_PG |
| **57** | 05-Sep-17 | Sottrum; Germany | Healthy | No | 4421 | B1 | commensal_PG |
| **58** | 05-Sep-17 | Sottrum; Germany | Healthy | No | 744 | A | commensal_PG |
| **59** | 05-Sep-17 | Sottrum; Germany | Healthy | No | 744 | A | commensal_PG |
| **60** | 05-Sep-17 | Sottrum; Germany | Healthy | No | 10 | A | commensal_PG |
| **61** | 05-Sep-17 | Sottrum; Germany | Healthy | No | 2137 | B1 | commensal_PG |
| **62** | 05-Sep-17 | Sottrum; Germany | Healthy | No | 4421 | B1 | commensal_PG |
| **63** | 05-Sep-17 | Sottrum; Germany | Healthy | No | 744 | A | commensal_PG |
| **64** | 05-Sep-17 | Sottrum; Germany | Healthy | No | 58 | B1 | commensal_PG |
| **65** | 05-Sep-17 | Sottrum; Germany | Healthy | No | 4421 | B1 | commensal_PG |
| **66** | 05-Sep-17 | Sottrum; Germany | Healthy | No | 4421 | B1 | commensal_PG |
| **67** | 05-Sep-17 | Sottrum; Germany | Healthy | No | 4421 | B1 | commensal_PG |
| **68** | 02-Aug-17 | Sottrum; Germany | Sick | No | 405 | D | pathogenic_PG |
| **69** | 28-Feb-17 | Sottrum; Germany | Healthy | No | 1001 | B1 | commensal_PG |
| **70** | 21-Mar-17 | Sottrum; Germany | Healthy | No | 1280 | F | pathogenic_PG |
| **72** | 26-Jun-17 | Sottrum; Germany | Healthy | No | 1001 | B1 | commensal_PG |
| **73** | 28-Apr-17 | Sottrum; Germany | Healthy | Yes | 362 | D | pathogenic_PG |
| **74** | 02-May-17 | Sottrum; Germany | Healthy | No | 362 | D | pathogenic_PG |
| **75** | 07-Oct-17 | Sottrum; Germany | Healthy | No | 1722 | F | pathogenic_PG |
| **77** | 07-Jan-17 | Sottrum; Germany | Healthy | No | 2325 | A | commensal_PG |
| **78** | 07-Oct-17 | Sottrum; Germany | Healthy | No | 1722 | F | pathogenic_PG |
| **79** | 07-Oct-17 | Sottrum; Germany | Healthy | No | 1146 | B1 | commensal_PG |
| **80** | 07-Oct-17 | Sottrum; Germany | Healthy | No | 1245 | E | commensal_PG |
| **81** | 07-Oct-17 | Sottrum; Germany | Healthy | No | 1722 | F | pathogenic_PG |
| **82** | 11-Jul-17 | Sottrum; Germany | Healthy | No | 10 | A | commensal_PG |
| **83** | 06-Nov-17 | Sottrum; Germany | Healthy | No | 1396 | B1 | commensal_PG |
| **84** | 13-Nov-17 | Sottrum; Germany | Healthy | Yes | 744 | A | commensal_PG |
| **85** | 13-Nov-17 | Sottrum; Germany | Healthy | Yes | 10 | A | commensal_PG |
| **86** | 06-Nov-17 | Sottrum; Germany | Sick | No | 73 | B2 | pathogenic_PG |
| **88** | 29-Dec-17 | Sottrum; Germany | Sick | No | 155 | B1 | commensal_PG |


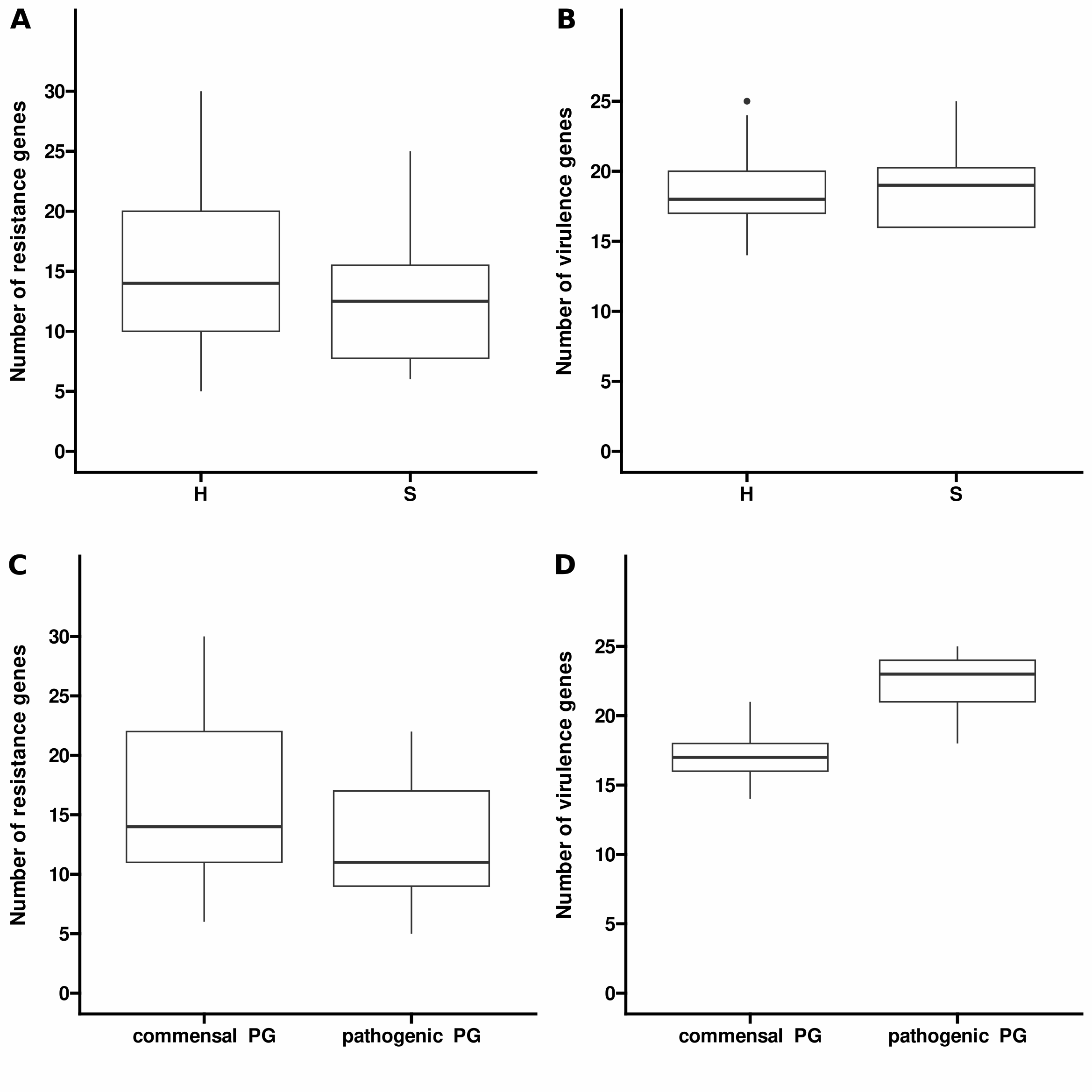


Fig. S1: Distribution of antimicrobial resistance and virulence associated genes within healthy and sick dogs as and commonly commensal phylogroups and pathogenic phylogroups. PG: phylogroup
